# Characterization of the miRNA regulators of the human ovulatory cascade

**DOI:** 10.1038/s41598-018-33807-y

**Published:** 2018-10-23

**Authors:** G. M. Yerushalmi, M. Salmon-Divon, L. Ophir, Y. Yung, M. Baum, G. Coticchio, R. Fadini, M. Mignini-Renzini, M. Dal Canto, R. Machtinger, E. Maman, A. Hourvitz

**Affiliations:** 1 Reproduction Lab and IVF Unit, Department of Obstetrics and Gynecology, Sheba Medical Center, 52662, Tel Hashomer, Affiliated with the Sackler Faculty of Medicine, Tel Aviv University, Tel Aviv, Israel; 2 Department of Molecular Biology, Ariel University, Ariel, Israel; 3 Biogenesi, Reproductive Medicine Centre, Istituti Clinici Zucchi, Via Zucchi 24, 20052 Monza, Italy

## Abstract

Ovarian follicular development and ovulation are complex and tightly regulated processes that involve regulation by microRNAs (miRNAs). We previously identified differentially expressed mRNAs between human cumulus granulosa cells (CGCs) from immature early antral follicles (germinal vesicle - GV) and mature preovulatory follicles (metaphase II - M2). In this study, we performed an integrated analysis of the transcriptome and miRNome in CGCs obtained from the GV cumulus-oocyte complex (COC) obtained from IVM and M2 COC obtained from IVF. A total of 43 differentially expressed miRNAs were identified. Using Ingenuity IPA analysis, we identified 7288 potential miRNA-regulated target genes. Two hundred thirty-four of these target genes were also found in our previously generated ovulatory gene library while exhibiting anti-correlated expression to the identified miRNAs. IPA pathway analysis suggested that miR-21 and *FOXM1* cooperatively inhibit *CDC25A*, *TOP2A* and *PRC1*. We identified a mechanism for the temporary inhibition of *VEGF* during ovulation by *TGFB1*, miR-16-5p and miR-34a-5p. The linkage bioinformatics analysis between the libraries of the coding genes from our preliminary study with the newly generated library of regulatory miRNAs provides us a comprehensive, integrated overview of the miRNA-mRNA co-regulatory networks that may play a key role in controlling post-transcriptomic regulation of the ovulatory process.

## Introduction

Ovarian follicular development and ovulation in mammals are highly complex and tightly regulated processes that involve the selection of a dominant follicle, reactivation of oocyte meiosis, rupture of the follicle wall, cumulus-oocyte expansion and tissue remodeling to form the corpus luteum. Oocyte growth and maturation are linked to the developmental properties of somatic cell populations in the expanding follicle as bi-directional signaling that occurs between oocytes and granulosa cells^1^. The genes involved in follicle maturation and ovulation are the subject of growing interest and have been well-studied by several laboratories, including ours^2,3^.

MicroRNAs (miRNAs) are small (~22-nucleotide), non-coding regulatory RNA molecules encoded by plants, animals and some viruses that regulate a variety of developmental and physiological processes (reviewed in^4,5^).

In the last few years, by virtue of the application of high-throughput sequencing techniques, it became clear that eukaryotes transcribe up to 90% of their genomic DNA. However, only 1-2% of these transcripts are protein-coding genes^6^.

Several studies have found specific miRNAs that target important ovarian molecules such as progesterone receptor (*PGR*)^7^, aromatase (*CYP19A1*), follicle stimulating hormone receptor (*FSHR*)^8^ and the *FYN* pathway^9^. miRNAs were suggested to function as RNA-based paracrine factors in the crosstalk between oocytes and support cells of the follicle^10^. Furthermore, the expression of a subset of miRNAs has been shown to vary during follicle/luteal development^11–13^. Effects on granulosa cell function and/or gene expression, largely in mice, have been reported for a handful of miRNAs, and these include effects on cell survival (miR-21 and miR-23a), proliferation (miR-145, miR-503, and miR-224), estradiol production (miR-224, miR-383, and miR-378) and terminal differentiation (miR-132 and miR-212) of cultured cells (reviewed in^14,15^).

In humans, miRNAs have already been profiled in cumulus versus mural granulosa cells^8^, Polycystic Ovary Syndrome (PCOS) patients^16^ and cumulus cells according to oocyte nuclear maturity^17^. miRNAs have been suggested as potential biomarkers in IVF^18^.

In the past several years, miRNA and mRNA integrated analysis (MMIA) has become a tool for examining the biological functions of miRNA expression. As the biological effects of miRNAs are due to their modulation of target RNA expression, accurate miRNA target prediction is essential to any study of miRNA function.

To understand the molecular and regulatory mechanisms necessary for follicular maturation and ovulation, we aimed to uncover the miRNA transcriptome involved in these processes. Recent advances in genomics allow a systematic approach to identify critical genes and regulated pathways involved in oocyte maturation and ovulation. Using whole transcriptome sequencing, we recently identified mRNAs that are differentially expressed between immature mid-antral follicles and mature pre-ovulatory follicles^3^. The resulting database provides unprecedented insight into the processes and pathways involved in follicular maturation and ovulation. To complete the identification of factors involved in the ovulatory process, the aim of this work was to generate a library of global miRNAs involved in this process and to link the new ovulatory miRNA library with the previously described mRNA library. This enabled us to identify new regulatory mechanisms responsible for the final follicular maturation and ovulatory processes.

## Results

### miRNA profile differences in compact and expanded cumulus cells

To elucidate the roles of miRNAs in human folliculogenesis and ovulation, we generated a library of regulated miRNAs during the final stage of follicular maturation and ovulation. We used NanoString on RNA extracted from two groups of cumulus granulosa cells: (1) Compact cumulus cells surrounding GV oocytes that were acquired during IVM treatment (CCGV *n* = 4) and (2) Expanded cumulus cells enclosing M2 oocytes that were acquired during IVF treatment (CCM2, *n* = 3). One CCGV sample was an outlier and was removed from further downstream analysis. Thus, the final number of samples in each group was three.

**Figure 1:**
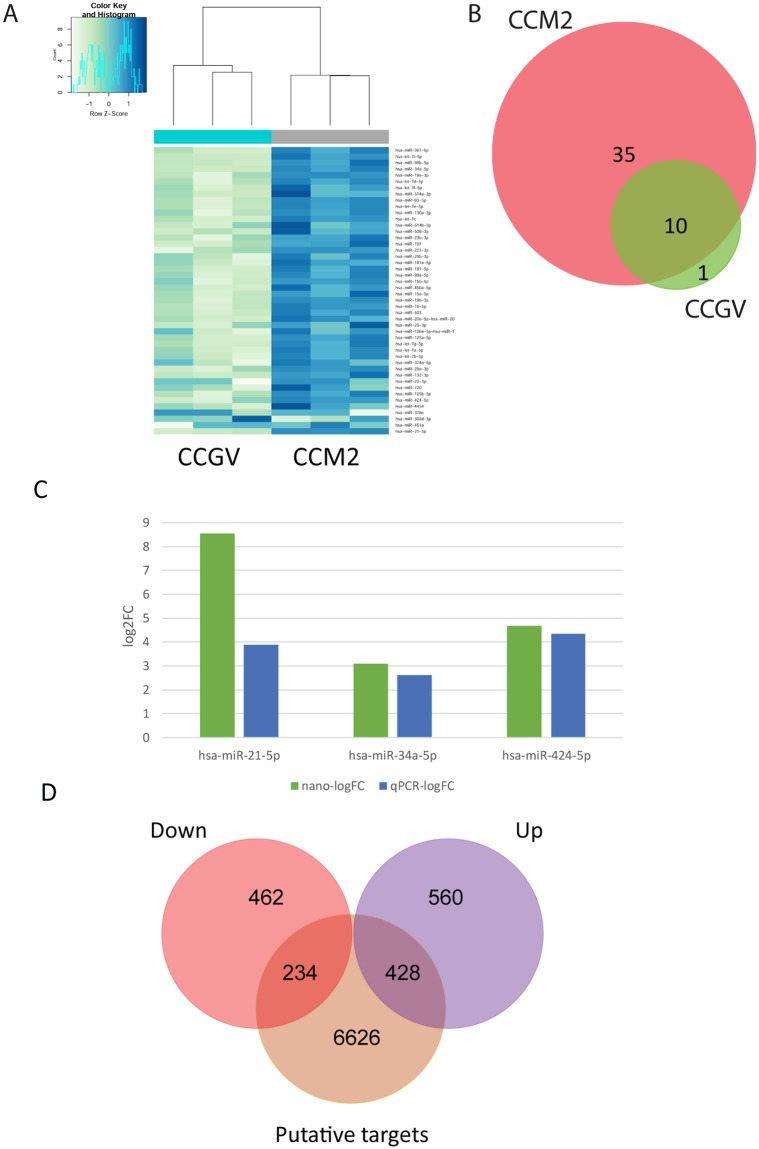
(**A**) Hierarchical clustering of CCGV (n = 3) and CCM2 (n = 3) samples based on miRNA expression levels. Each column represents a sample and each row represents a transcript. Expression level of each miRNA in a single sample is depicted according to the color scale. (**B**) Venn diagram of selective and co-expressed miRs in CCGV and CCM2 samples. A total of 45 miRs were expressed in CCM2 (pink) and 11 miRs were expressed in CCGV (green). Most notably, 35 miRs were exclusively expressed in the IVF group and 10 miRs were shared by both samples. (**C**) Total mRNA was purified from CCs denuded from GV COC aspirated during IVM procedures (CCGV) and CCs denuded from M2 COC (CCM2) aspirated during IVF procedures. The miRNAs were subjected to qPCR in duplicate with the examined genes and RNU6B primers. Gene expression was calculated relative to the RNU6B level in the same sample and expression levels were compared using Student’s t-test. The difference reached a level of significance (P < 0.05) for all tested genes. NanoString (green) and qPCR (blue) results are presented as log2-fold change between CCM2 and CCGV samples. (**D**) Venn diagram showing the relationship between the putative miRNA targets (brown) and experimentally differentially expressed mRNAs (from Yerushaslmi *et al*.^3^, upregulated mRNA (purple), downregulated mRNA (red)). Of all the putative miRNA targets, 234 were negatively correlated.

A Venn diagram of these groups is shown in Fig. 1B, illustrating the numbers of selective and co-expressed miRNAs in the two conditions, CCGV and CCM2. A total of 45 miRNAs were detected in CCM2 and 11 miRNAs were detected in CCGV. Most notably, 35 miRNAs were exclusively detected in the CCM2 group; only 1 was exclusively detected in the CCGV group and 10 miRNAs were shared between the two conditions (Table 1). hsa-miR-378e was exclusively expressed in the CCGV group, although the differential expression level was not statistically significant.

**Table 1:** miRNAs identified by the NanoString nCounter miRNA expression assay in human cumulus granulosa cells. Forty-three of the miRNAs were differentially expressed with a fold change >2 and an FDR < 5%.

miR	logFC CCM2 vs. CCGV	Average Expression CCGV	Average Expression CCM2	p-value	Adj. p-value	Putative granulosa cell function	References
hsa-let-7a-5p*	3.81363	3642.69	43742.2	0.000154	0.000256	Apoptosis, cumulus vs. mural	^8, 50, 63^
hsa-let-7b-5p	3.30288	2591.97	20276.4	0.000497	0.000693		
hsa-let-7c	3.87105	506.408	6725.24	3.53E-06	2.91E-05		
hsa-let-7d-5p	3.10472	376.498	2855.3	3.91E-05	0.000106		
hsa-let-7e-5p	3.54614	491.637	5066.29	1.15E-05	4.41E-05		
hsa-let-7f-5p*	2.66036	348.055	2230.98	0.000156	0.000256		
hsa-let-7g-5p*	4.17388	1762.22	31204.5	1.99E-05	6.11E-05		
hsa-let-7i-5p	3.2881	279.589	2871.92	7.02E-06	0.000036		
hsa-miR-106a-5p/ miR-17-5p	1.89582	1418.37	4268.29	0.017636	0.019316	Bovine follicle development	^64^
hsa-miR-107	2.20654	289.935	1226.41	0.000389	0.00056	Bovine follicle development, Steroidogenesis	^65, 66^
hsa-miR-125a-5p	3.81061	2536.53	30936.3	9.05E-05	0.000189	Apoptosis	^67^
hsa-miR-125b-5p	5.00927	2516.64	70782.3	3.79E-06	2.91E-05	Bovine follicle development, Steroidogenesis, cumulus vs. mural	^8, 65, 66^
hsa-miR-130a-3p	3.27081	559.043	4177.33	5.68E-05	0.000131	Bovine follicle development, PCOS	^66, 68^
hsa-miR-132-3p	5.59724	290.378	15338.4	3.9E-09	8.96E-08	cumulus vs. mural, Steroidogenesis, PCOS, murine follicle development	^8, 11, 68^
hsa-miR-15a-5p	3.15798	1280.84	10868	0.000342	0.000507	Steroidogenesis	^65^
hsa-miR-15b-5p	2.42639	1952.83	8482.74	0.004694	0.005682	Steroidogenesis	^65^
hsa-miR-16-5p	3.5448	1493.38	16847.5	0.000103	0.000206	Bovine follicle development	^8^
hsa-miR-181a-5p	3.82643	404.396	5005.58	7.08E-06	0.000036	cumulus vs. mural, proliferation	^8, 69^
hsa-miR-191-5p*	2.80069	1557.66	10258.1	0.001191	0.001565	Bovine follicle development, cumulus vs. mural	^8, 70^
hsa-miR-19a-3p	3.09878	381.085	2980.11	4.71E-05	0.000114	Bovine follicle development, Steroidogenesis, PCOS	^64– 66, 68^
hsa-miR-19b-3p	3.33449	1177.47	10517.6	0.000143	0.000253	Ovarian hyperstimulation	^71^
hsa-miR-20a-5p/ miR-20b-5p	3.85536	1094.21	16458.7	2.87E-05	8.26E-05	cumulus vs. mural	^8^
hsa-miR-21-5p*	8.54162	21.5677	6740.8	1.69E-11	7.79E-10	cumulus vs. mural, murine follicle development, apoptosis	^8, 11, 40^
hsa-miR-223-3p	2.58013	254.152	1404.38	0.000114	0.000216	cumulus vs. mural, Steroidogenesis, PCOS	^8, 72^
hsa-miR-22-3p*	1.75262	1807.13	4565.75	0.041854	0.044774	cumulus vs. mural, Steroidogenesis, PCOS	^8, 72^
hsa-miR-23b-3p	2.59206	281.984	1384.31	0.000117	0.000216	Bovine ovary	^73^
hsa-miR-25-3p	2.06898	1683.34	7156.33	0.013105	0.014703	Steroidogenesis	^65^
hsa-miR-29a-3p	4.94371	536.265	13587.1	5.05E-07	7.74E-06	Steroidogenesis	^74^
hsa-miR-29b-3p	3.91754	672.73	7187.08	0.000016	5.26E-05	Steroidogenesis, cumulus vs. mural	^8, 65^
hsa-miR-302d-3p	−0.25	1660.7	1305.76	0.710	0.710**	n/a	
hsa-miR-34a-5p	3.09029	245.28	2018.34	8.49E-06	0.000036	Apoptosis	^55, 75^
hsa-miR-361-5p	3.15727	329.908	2675.28	1.59E-05	5.26E-05	Murine follicle development	^11^
hsa-miR-374a-5p	2.05355	841.464	2757.13	0.007376	0.008482	cumulus vs. mural,	^8^
hsa-miR-378e	−1.3021	3643.64	2397.74	0.13083	0.13678**	Steroidogenesis	^76^
hsa-miR-424-5p	4.68931	1405.72	27294.5	7.48E-06	0.000036	Age	^17^
hsa-miR-4454	3.79594	2852.29	62869.6	0.000325	0.000498	n/a	
hsa-miR-450a-5p	2.70382	1349.62	8663.35	0.001472	0.001881	Bovine follicle development	^66^
hsa-miR-451a*	1.218	1669.72	3424.96	0.176	0.180**	cumulus vs. mural	^8^
hsa-miR-503	4.2726	750.23	13810.1	2.66E-06	2.91E-05	Bovine follicle development,	^12, 68, 77^
hsa-miR-508-3p	3.27969	208.958	2839.37	8.61E-06	0.000036	PCOS	^16^
hsa-miR-514a-3p	2.63133	282.897	2477.97	0.000185	0.000294	PCOS	^16^
hsa-miR-514b-5p	1.8413	431.937	1844.75	0.005467	0.006449	PCOS	^72^
hsa-miR-720	3.10848	1958.04	22615.3	0.001832	0.002277	PCOS, Age	^16, 17^
hsa-miR-93-5p	3.30979	620.555	5549.2	0.000045	0.000114	Proliferation, PCOS	^78, 79^
hsa-miR-99a-5p*	2.89148	1585.53	10215.6	0.000935	0.001266	cumulus vs. mural, Bovine follicle development	^8, 70, 80^
hsa-miR-99b-5p	2.74138	357.19	2252.67	7.72E-05	0.000169	Age, Bovine follicle development, PCOS	^64, 67, 81^

*miRNA detected as one of the 10 most abundant in human cumulus cells^8^.

**Differential expression between CCGV and CCM2 samples was not significant.

With NanoString data inspected, all quality control parameters were within the acceptable ranges. Unsupervised hierarchical cluster analysis based on the expression of all detected miRNAs shows that the biological replicates cluster to their corresponding groups (Fig. 1A).

Almost all of the expressed miRNAs were significantly differentially expressed (43 of the 46 identified miRNAs) between CCGV and CCM2, with a fold change >2 and a false discovery rate (FDR) < 5%. Of these, 25 represent unique seed sequences. Our results indicate that all differentially expressed miRNAs were up-regulated in CCM2 cells.

### Validation of selected miRNA expression levels as estimated by qPCR

We used qPCR analysis to validate the results obtained by NanoString. We selected three miRNAs that were identified as differentially expressed by NanoString. qPCR was performed in three different experiments, as shown as fold induction in Fig. 1C, and demonstrated agreement with NanoString relative expression data.

### Identification of differentially expressed miRNA target genes – *in silico* analysis

Because miRNAs act by regulating the expression of target genes, precise miRNA target prediction is important for elucidating miRNA function. The identification of miRNA target genes was performed using the microRNA target filter tool of QIAGEN’s Ingenuity Pathway Analysis software, taking into account only experimentally validated and highly confident predictions of miRNA target interactions. We found 7288 putative miRNA target genes of which 727 (294 unique) were experimentally validated (Supp. Tables S3 and S4).

### Identification of differentially expressed miRNAs that negatively correlated with ovulatory target genes - experimental analysis

To further dissect the putative ovulatory miRNA targets, we combined our previously generated mRNA library^3^ and miRNA expression data using the microRNA target filter tool of QIAGEN’s Ingenuity Pathway Analysis^19^. A Venn diagram depicting the relationships between the putative miRNA targets and experimentally differentially expressed mRNAs is depicted in Fig. 1D.

We chose to focus on negatively correlated targets. Since all of the differentially expressed CCM2 miRNAs were upregulated, all chosen paired mRNA targets were downregulated. The 696 downregulated mRNAs in the CCM2 group (compared to CCGV group) were paired with the 7288 putative miRNA targets. Of the 696 downregulated mRNAs, 234 were targets of 22 differentially expressed miRNAs (of the 25 having unique seed sequences). In total, these 234 genes formed 479 miRNA-target gene pairs with an inverse correlation of expression (Table 2).

**Table 2:** The 234 miRNA target genes that negatively correlated with the expression of 22 differentially expressed miRNAs.

ID	Symbol	Count of Targets	Target mRNA
hsa-let-7g-5p	let-7a-5p (and other miRNAs w/seed GAGGUAG)	40	ADAMTS15, ANGPTL2, AURKB, B3GAT1, C15orf39, CDC25A, CHRD, CMTM6, DAPK1, DSP, ESPL1, ESR2, ETNK2, EZH2, FANCD2, FRMD4B, GAS7, GATM, IFNLR1, KIF21B, LINGO1, MMP11, MYO5B, MYRIP, NEMP1, NOS1, PAG1, PARM1, PLXNC1, PPT2, PRIM1, RBM38, RIMS3, RRM2, SLC1A4, SLC37A4, THBS1, TMPO, TYMS, UNC5A
hsa-miR-107	miR-103-3p (and other miRNAs w/seed GCAGCAU)	26	ADGRB3, AJUBA, BCL11A, CDCA4, CLSPN, ESR1, HOXD10, HSDL1, IGSF3, IHH, LRP1, MBOAT1, NAV1, NEIL1, NOS1, NRP2, OLFM1, PAG1, RIMS3, RNF19A, RTKN2, S1PR3, SOWAHC, SYNDIG1, TMEM35, WHSC1
hsa-miR-125b-5p	miR-125b-5p (and other miRNAs w/seed CCCUGAG)	29	ADAMTS15, AJUBA, C15orf39, CACNB2, CDC25A, ENTPD1, FAM78A, GLB1L2, HAPLN1, HCN1, HCN4, HOXD9, KCNH3, KCNIP3, LOXL1, MMP11, NEMP1, NUP210, OPALIN, PARM1, PPT2, PRSS35, RBM38, SLC4A8, ST6GAL1, STMN3, TMCC2, TNFSF4, VEGFA
hsa-miR-130a-3p	miR-130a-3p (and other miRNAs w/seed AGUGCAA)	32	ADAMTS18, ADCY2, ADGRB3, ARX, BCL11A, CEP55, CHST1, DEPDC1, DIAPH3, ESCO2, ESR1, FAM78A, GSE1, HES1, IGSF3, INHBB, ITPKB, KLHDC8A, SIK1, MB21D2, MTCL1, MYO1D, NRP2, PLCL2, PLLP, PRR15, RALGAPA2, SHANK2, SOX4, SOX5, ST8SIA5, ZEB1
hsa-miR-132-3p	miR-132-3p (and other miRNAs w/seed AACAGUC)	12	ARX, COL4A4, HAPLN1, HUNK, ITPKB, KIF21B, OLFM1, PALM2, RAP2B, SHANK2, SOX4, SOX5
hsa-miR-424-5p	miR-16-5p (and other miRNAs w/seed AGCAGCA)	39	ADAMTS18, C14orf37, CASR, CD47, CDC25A, CDCA4, CHEK1, CLSPN, CRHBP, DPH5, GLCE, GSE1, HCN1, HERC6, IHH, LY6E, MARCH4, MCF2L, MSH2, MYO5B, MYRIP, NAV1, NOS1, NRP2, NUP210, PAG1, PARM1, PLXNA2, PPIF, PPT2, PRIM1, RIMS3, SLC4A8, SOWAHC, SOX5, SYNDIG1, VEGFA, WHSC1, ZNF423
hsa-miR-20a-5p	miR-17-5p (and other miRNAs w/seed AAAGUGC)	36	AJUBA, CDC25A, CEP128, CHAF1A, CNNM3, DDIAS, DPF3, E2F1, ELAVL2, ESR1, FRMD4B, GUCY1A3, HAUS8, HCN4, ITPKB, SIK1, MARCH4, MCF2L, MCM3, MYLIP, MYO1D, MYO5B, NR4A3, NRP2, PBK, POLQ, PRR15, RASL11B, RRM2, SEMA7A, SHANK2, SORL1, SOWAHC, SOX4, VEGFA, WHSC1
hsa-miR-181a-5p	miR-181a-5p (and other miRNAs w/seed ACAUUCA)	35	ACAN, ADAMTS18, ADGRB3, ATP1B1, ELAVL2, ERG, ESR1, FAM19A2, GAL3ST3, GAS7, GREM1, GSE1, HAPLN1, HCN1, HEY2, HMGB2, MB21D2, MBOAT1, MTCL1, NR4A3, PAG1, PALM2, PARM1, PEG3, PLCL2, POLQ, PPP1R12B, RALGAPA2, RFTN2, RTKN2, SFRP4, SOX5, THBS2, UNC5A, WHSC1
hsa-miR-191-5p	miR-191-5p (and other miRNAs w/seed AACGGAA)	2	BCL11A, SOX4
hsa-miR-19b-3p	miR-19b-3p (and other miRNAs w/seed GUGCAAA)	36	ARHGAP11A, CEP55, CHST1, DAAM1, DAG1, EDARADD, ELAVL2, ENC1, ESR1, IFI44L, IGSF3, INHBB, ITPKB, PCLAF, MATN2, MATN3, MB21D2, MTCL1, MYLIP, NAV1, NRP2, PARM1, PLCL2, PLXNC1, PRC1, RAP2B, RNF19A, SHANK2, SOGA1, SORL1, SOX4, SOX5, SPTSSB, SYNPO2, THBS1
hsa-miR-21-5p	miR-21-5p (and other miRNAs w/seed AGCUUAU)	9	BCL11A, CDC25A, DAG1, ERG, MATN2, MSH2, PAG1, SOX5, ST6GAL1
hsa-miR-22-3p	miR-22-3p (miRNAs w/seed AGCUGCC)	10	ADCK2, CHGA, DERL3, EMILIN3, ESR1, GATM, HUNK, NUSAP1, RAPGEF3, VIT
hsa-miR-223-3p	miR-223-3p (miRNAs w/seed GUCAGUU)	14	ADGRB3, ATP1B1, CSPG5, CTSV, E2F1, ECT2, GALNT18, LAYN, MYO5B, NUP210, OLFM1, RIMS3, STMN1, ZEB1
hsa-miR-23b-3p	miR-23a-3p (and other miRNAs w/seed UCACAUU)	41	ARHGEF6, BCL11A, CDC6, COL4A4, DAPK1, DDAH1, DEPDC1, DTL, EDARADD, ENC1, EXOC3L4, FANCI, FSHR, GALNT12, GLCE, GREM1, HAPLN1, HES1, HMGB2, HOXD10, IHH, KCNIP4, MAP7D2, MKX, NDC1, NEMP1, PDGFA, PLXNC1, PPIF, PRSS35, RAD51AP1, RAP2B, SFRP4, SOWAHC, SPTSSB, SYNPO2, TMPO, TOP2A, WHSC1, ZEB1, ZNF423
hsa-miR-29a-3p	miR-29b-3p (and other miRNAs w/seed AGCACCA)	29	ADAMTS18, ADAMTS2, ADCYAP1R1, AGPAT4, ATP1B1, AUNIP, BCL11A, CLEC2L, COL4A4, CSPG4, GAS7, HAPLN1, KIF24, LPL, MAP2K6, MFAP2, MYBL2, NASP, NAV1, NLGN3, PAG1, PALM2, PDGFC, RNF19A, RTKN2, SLC16A1, SOWAHC, TMEM132A, VEGFA
hsa-miR-34a-5p	miR-34a-5p (and other miRNAs w/seed GGCAGUG)	25	ABLIM1, ADCY5, ANK2, CD47, CDC25A, COL4A4, DAAM1, FAM107A, GABRA3, GLCE, INHBB, MYRIP, NAV1, NOS1, PAG1, PALM2, PTGIS, RIMS3, SDK2, SLCO3A1, SOGA1, SOX4, TMEM35, VEGFA, WHSC1
hsa-miR-361-5p	miR-361-5p (miRNAs w/seed UAUCAGA)	7	ADCY2, ERG, FOXM1, GCOM1, PEG3, VEGFA, VWDE
hsa-miR-374a-5p	miR-374b-5p (and other miRNAs w/seed UAUAAUA)	21	CD47, FAM169A, FAM19A2, GAS7, HAPLN1, HES1, HUNK, INHBB, MAP2K6, MKX, MMRN1, NR4A3, PLXNA2, RTKN2, SHANK2, SNTB1, SOX4, SPTSSB, UST, VEGFA, ZNF423
hsa-miR-503	miR-503-5p (miRNAs w/seed AGCAGCG)	14	CASR, CDC25A, CDCA4, CHEK1, CNNM3, DNAAF3, GLCE, IHH, NAV1, NOS1, PARM1, SOX5, VEGFA, ZNF423
hsa-miR-514b-5p	miR-513c-5p (and other miRNAs w/seed UCUCAAG)	2	BRCA2, PDE6A
hsa-miR-514a-3p	miR-514a-3p (and other miRNAs w/seed UUGACAC)	1	PEG3
hsa-miR-25-3p	miR-92a-3p (and other miRNAs w/seed AUUGCAC)	19	ACAN, ADGRB3, ANGPTL2, BCL11A, CHGA, CHST1, DAG1, GLCE, GRIA1, HAPLN1, HOXD10, SIK1, MARCH4, MYLIP, NR4A3, PALM2, SORL1, SOX4, SYNDIG1

To understand the putative roles of these miRNA target genes, two lists of putative regulated genes were generated. The first list included all of the putative miRNA targets (*in silico* analysis) and the second list included only the negatively correlated differentially expressed targets (experimental analysis). This contributes to both a broad and targeted view of the ontologies and pathways related to the roles of miRNAs in ovulation. Gene Ontology (GO) and pathway analyses were performed using the GeneAnalytics tool^20^.

### Gene Ontology and pathway classification of the *in silico* analysis of miRNA target genes

**Figure 2:**
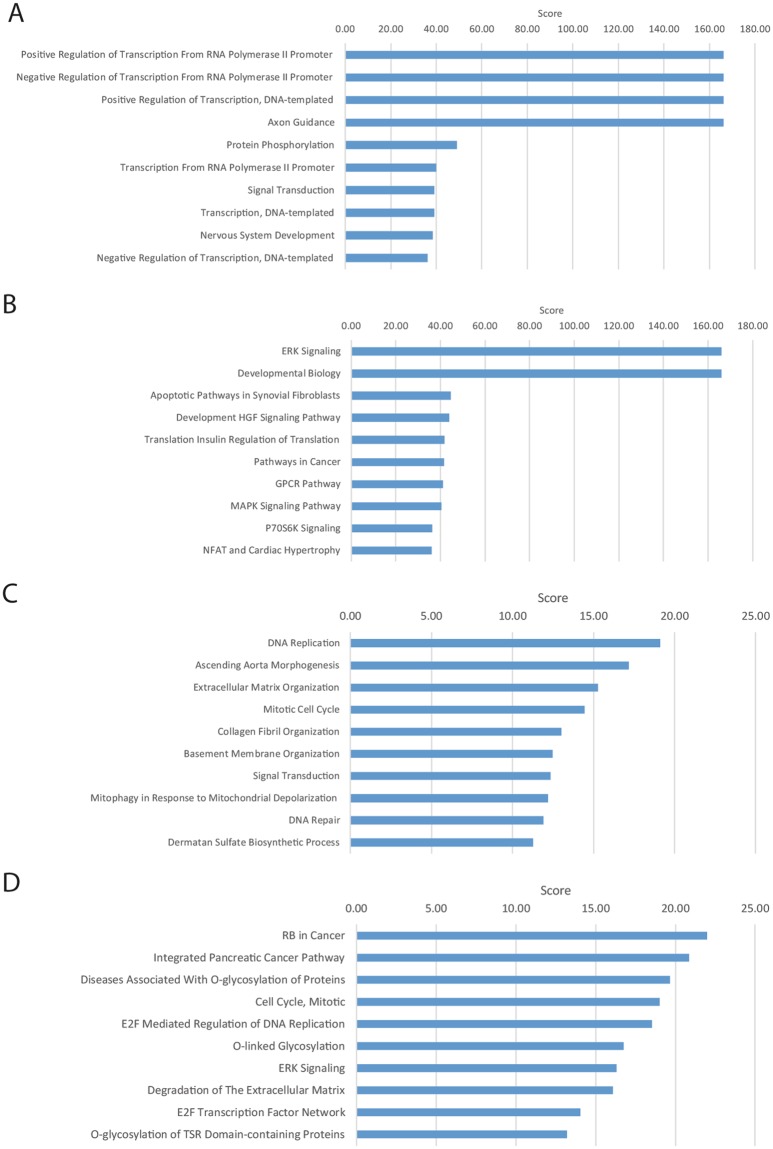
GeneAnalytics analysis of all 7288 putative miRNA targets (**A**,**B**) and of the 234 negatively correlated differentially expressed miRNA targets (**C**,**D**). Presented are the top 10 enriched GO terms (**A**,**C**) and the top 10 enriched pathways (**B**,**D**). The presented GeneAnalytics score is a transformation (−log2) of the p-value; hence, higher scores represent stronger enrichment.

For the unfiltered miRNA targets (7288 genes), we identified 78 high-score GO terms (Fig. 2A and Supp. Table S5). The GO terms included 9 “transcription regulation” ontologies and 15 “signaling” ontologies, including neurotropin TRK signaling, FGF receptor signaling, EGF receptor signaling, TGFB receptor signaling, WNT signaling, VEGF signaling, insulin receptor signaling and RAS signaling. Other important processes included axon guidance, nervous system development, negative regulation of cell proliferation, *in utero* embryonic development and angiogenesis and cell migration.

We identified 144 high-score pathways that included 67 signaling pathways (Fig. 2B and Supp. Table S6). The top scoring signaling pathways were ERK signaling, HGF signaling, GPCR signaling, MAPK signaling, P70-S6K signaling and RAS signaling. Other pathways included NFAT and cardiac hypertrophy, proteoglycans in cancer, focal adhesion, endocytosis and axon guidance.

### Gene Ontology and pathway classification of the negatively correlated miRNA target genes

For the subset of negatively correlated miRNA targets (234 genes), only four high scoring and four medium scoring GO terms were identified. The GO terms included DNA replication, ascending aorta morphogenesis, extracellular matrix organization and mitotic cell cycle. The top medium scoring GO terms included collagen fibril, basement membrane organization and signal transduction (Fig. 2C and Supp. Table S7). The nine high scoring pathways included RB in cancer, integrated pancreatic cancer pathway, diseases associated with O-glycosylation, cell cycle, E2F-mediated DNA replication, degradation of extracellular matrix and ERK signaling (Fig. 2D and Supp. Table S8).

### Upstream regulator analysis of negatively correlated miRNA target genes

We used Ingenuity’s IPA upstream regulator analytic feature to identify a cascade of upstream transcriptional regulators that can explain the observed gene expression changes of the negatively correlated genes beyond the identified miRNAs. These regulators can be transcription factors (TFs) and any gene or small molecule that has been observed experimentally to affect gene expression. TF and miRNA may mutually regulate each another or regulate a shared target gene and, as such, enhance the robustness of gene regulation. Hence, TF-miRNA regulatory network analysis will be helpful to decipher gene expression regulatory mechanisms of the ovulatory process.

A total of 68 significant upstream regulators were identified (Z-score > ±2, Supp. Table S9). The top ten inhibited regulators and activated regulators are presented in Tables 3 and 4, respectively.

**Table 3:** Top 10 inhibited upstream regulators of negatively correlated miRNAs and targets identified by ingenuity IPA analysis^19^.

Upstream Regulator	Exp Log Ratio	Molecule Type	Activation z-score	p-value of overlap	Target molecules in dataset
TGFB1		growth factor	−4.118	5.51E-07	ACAN, ADAMTS2, CDC25A, CSPG4, DAAM1, DAPK1, DSP, E2F1, ESPL1, ESR2, FAM107A, GAS7, GATM, GLCE, GREM1, GRIA1, GSE1, HES1, INHBB, LOXL1, LPL, MFAP2, MMP11, MYBL2, NR4A3, PDGFA, PLXNC1, PRC1, PRIM1, RAD51AP1, RAPGEF3, RASL11B, S1PR3, SEMA7A, SOX4, THBS1, TOP2A, TYMS, UST, VEGFA, ZEB1
MITF		transcription regulator	−3.606	3.85E-07	ACAN, AURKB, CEP55, CHAF1A, DAPK1, ECT2, ESPL1, FRMD4B, HAPLN1, HAUS8, HES1, ITPKB, SOX5, TMCC2
CSF2		cytokine	−3.448	1.48E-03	CHAF1A, FOXM1, HAUS8, IFNLR1, MCM3, NUSAP1, PPIF, PRC1, RRM2, SNTB1, STMN1, THBS1, TOP2A
MYC		transcription regulator	−3.281	9.61E-04	ACAN, AURKB, CD47, CDC25A, CHEK1, CSPG4, CTSV, DSP, E2F1, EZH2, FOXM1, HAPLN1, HES1, MSH2, PEG3, RRM2, SLC16A1, SOX5, STMN1, THBS1, THBS2, TYMS, VEGFA
FOXM1	−4.219	transcription regulator	−3.088	6.30E-07	AURKB, CDC25A, ESR1, FOXM1, PDGFA, PRC1, STMN1, TOP2A, VEGFA, ZEB1
HGF		growth factor	−2.925	1.12E-03	ANGPTL2, AURKB, CDC25A, CDC6, ENTPD1, FOXM1, HES1, NR4A3, PDGFA, PLXNA2, PRC1, THBS1, VEGFA, ZEB1
SP1		transcription regulator	−2.918	6.72E-03	CHGA, E2F1, ESR1, FOXM1, GRIA1, LPL, MMP11, MYBL2, NOS1, PDGFA, PDGFC, TYMS, VEGFA
CCND1		transcription regulator	−2.884	2.97E-07	CDC6, CEP55, CLSPN, DDIAS, DEPDC1, DTL, E2F1, ESCO2, FOXM1, KIAA0101, MYRIP, RRM2, SOX4, TYMS, ZNF423
Vegf		group	−2.834	3.46E-05	ACAN, ANGPTL2, AURKB, CDC25A, CDC6, DPF3, ENTPD1, FOXM1, HES1, IHH, INHBB, NR4A3, PDGFA, PLXNA2, PRC1, VEGFA, ZEB1
PTGER2		g-protein coupled receptor	−2.828	1.97E-05	CEP55, DEPDC1, ECT2, NUSAP1, PBK, PRC1, THBS1, VEGFA

**Table 4:** Top 10 activated upstream regulators of negatively correlated miRNAs and targets identified by ingenuity IPA analysis^19^.

Upstream Regulator	Exp Log Ratio	Molecule Type	Activation z-score	p-value of overlap	Target molecules in dataset
let-7		microrna	3.246	1.97E-05	AURKB,BRCA2, CDC25A, CDC6, CHEK1, EZH2, FANCD2, MCM3, PPP1R12B, RRM2, THBS1
mir-21	8.541619	microrna	3.093	3.46E-05	CDC25A, DDAH1, ECT2, MSH2, NUSAP1, PBK, PRC1, RAD51AP1, STMN1, TOP2A
RBL1		transcription regulator	2.923	3.79E-10	AURKB, CDC25A, CDC6, E2F1, HES1, MCM3, MYBL2, RRM2, THBS1, TYMS, ZEB1
CDKN2A		transcription regulator	2.837	1.04E-07	AURKB, CDC25A, CDCA4, CHAF1A, E2F1, EZH2, GAS7, HMGB2, HUNK, MYBL2, PDGFA, PEG3, RAD51AP1, RRM2, TMPO, VEGFA
BNIP3L		other	2.828	1.48E-07	CD47, CHEK1, E2F1, FANCD2, MYBL2, PRIM1, RRM2, TOP2A
miR-16-5p	4.689	mature microrna	2.777	1.71E-03	CDC25A, CHEK1, CRHBP, HERC6, MSH2, PPIF, PRIM1, VEGFA
let-7a-5p	4.174	mature microrna	2.768	2.61E-04	AURKB, CDC25A, DSP, FANCD2, PRIM1, SLC1A4, THBS1, TYMS
LY294002		chemical - kinase inhibitor	2.73	1.25E-03	ACAN, BRCA2, ESR1, HAPLN1, HES1, HMGB2, INHBB, LOC102724428/SIK1, NR4A3, THBS1, TOP2A, TYMS, VEGFA, ZEB1
CDKN1A		kinase	2.605	4.66E-12	AURKB, CDC25A, CDC6, CEP55, CHEK1, DTL, FANCI, FOXM1, HMGB2, KIAA0101, MCM3, MYBL2, NUSAP1, PBK, PRC1, STMN1, TOP2A, TYMS, VEGFA
sirolimus		chemical drug	2.527	2.84E-02	CDC25A, CHEK1, E2F1, GRIA1, NR4A3, STMN1, TMPO, TOP2A, TYMS, VEGFA

As expected, among the activated regulators, we identified 5 miRNAs, 3 of which were also differentially expressed in our analysis (Supp. Table S9).

**Figure 3:**
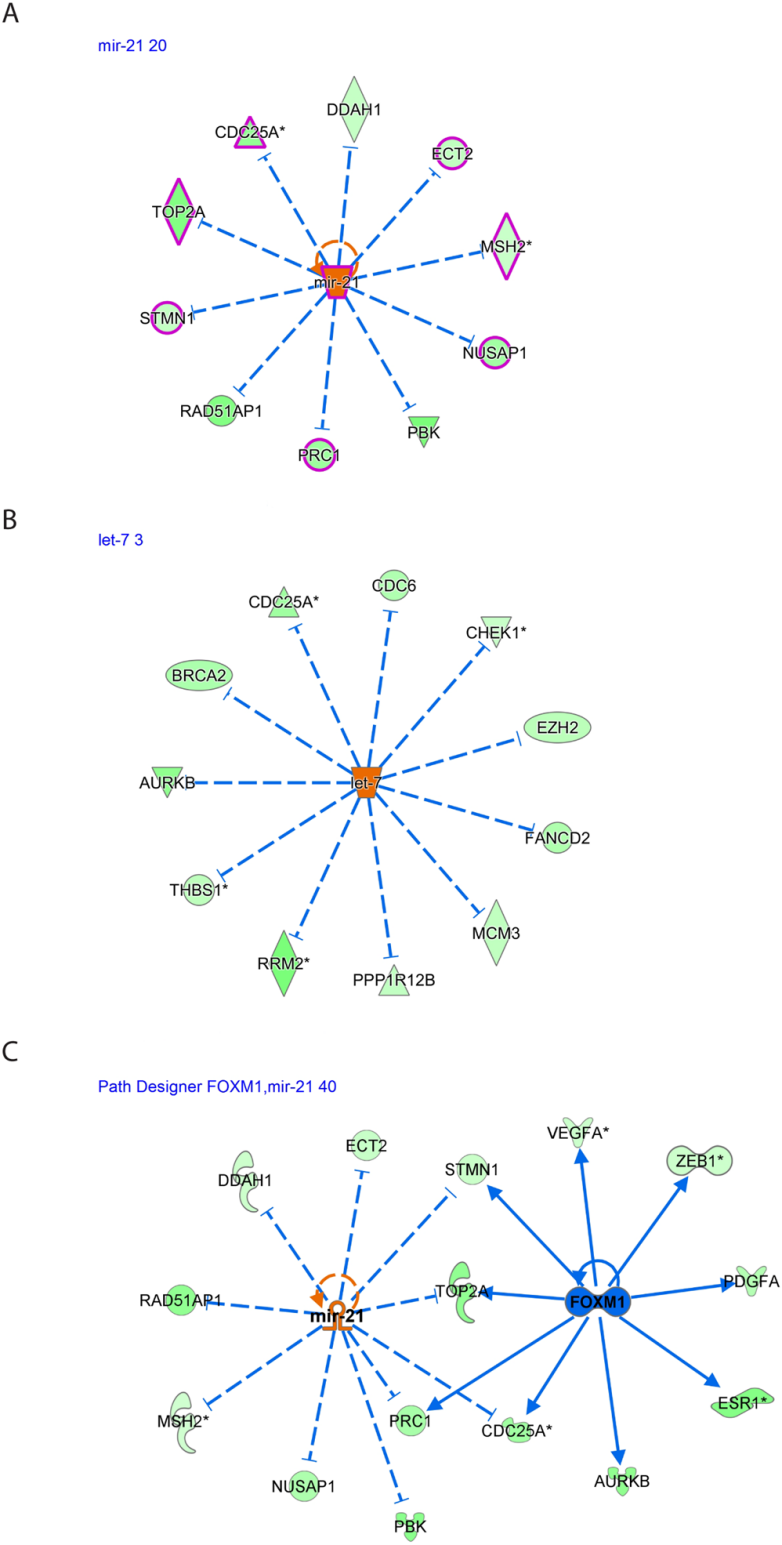
The resultant DE miRNAs were analyzed through the use of IPA (Ingenuity Pathway Analysis, QIAGEN Inc., https://www.qiagenbioinformatics.com/products/ingenuitypathway-analysis)^19^ to explore the inversely correlated miRNA-regulated pathways and mRNA expression in preovulatory granulosa cells. This analysis revealed that both (**A**) miR-21 and (**B**) let-7 are involved in the downregulation of several important genes. (**C**) Putative crosstalk between the *FOXM1* signaling pathway and miR-21 signaling. A number of genes and pathways are reciprocally regulated by these negatively correlated transcription regulators. mir-21 expression is upregulated, whereas *FOXM1* expression is downregulated during the ovulatory process.

We plotted several of the upstream regulators and their targets, as shown in Fig. 3. We identified miR-21 to be involved in the regulation of *CDC25A*, *DDAH1*, *ECT2*, *MSH2*, *NUSAP1*, *PBK*, *PRC1*, *RAD51AP1*, *STMN1* and *TOP2A* (Fig. 3A). Interestingly, most genes are involved in reproductive disease (highlighted in pink).

We identified let-7 to be involved in the regulation of *AURKB*, *BRCA2*, *CDC25A*, *CDC6*, *CHEK1*, *EZH2*, *FANCD2*, *MCM3*, *PPP1R12B*, *RRM2* and *THBS1* (Fig. 3B).

Among the notable interactions found was the reciprocal crosstalk between miR-21 (activated) and *FOXM1* (inhibited) signaling. Both transcriptional regulators inhibit *STMN1*, *TOP2A*, *CDC25A* and *PRC1* (Fig. 3C).

**Figure 4:**
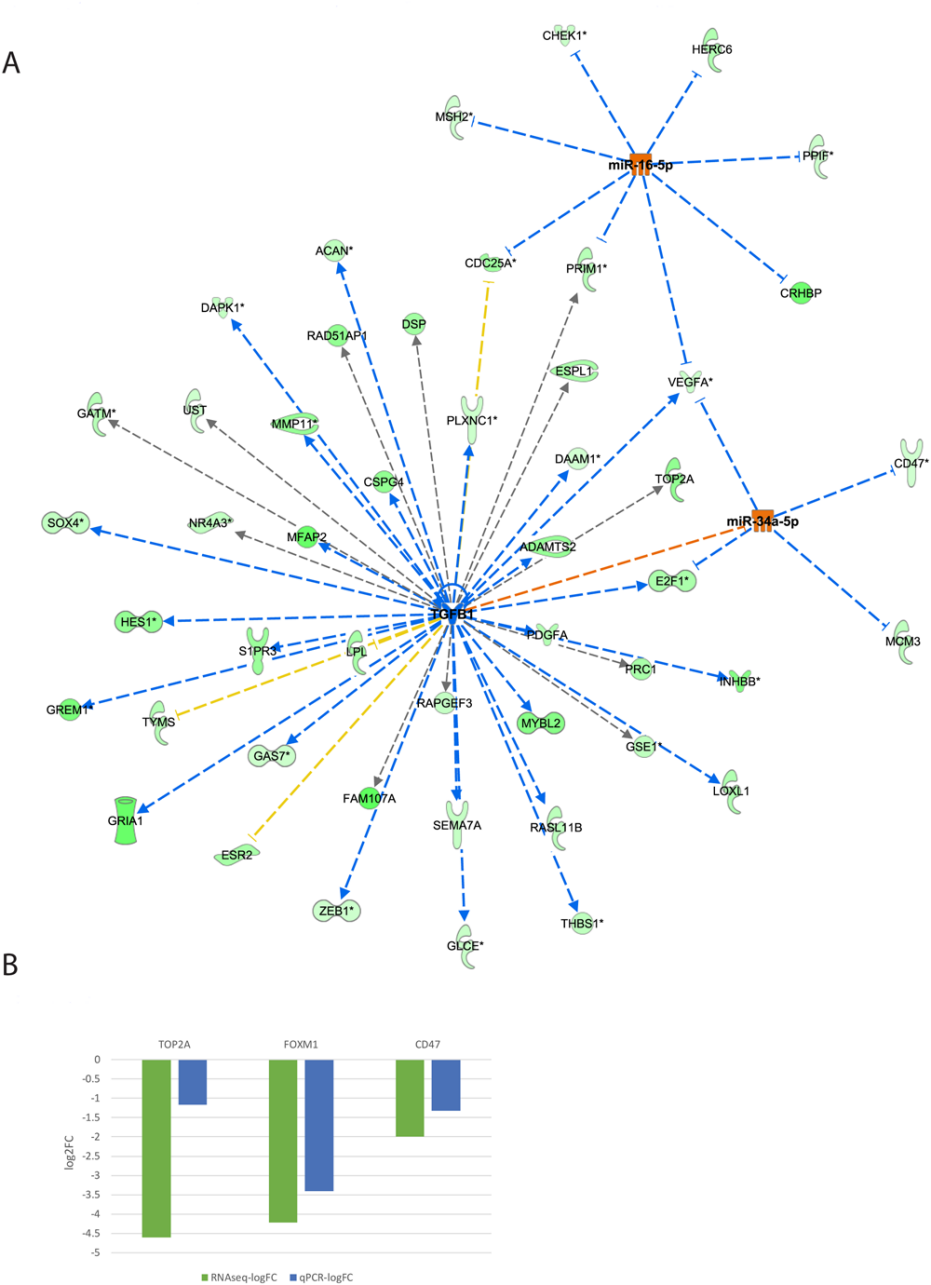
(**A**) The resultant DE miRNAs were analyzed through the use of IPA (Ingenuity Pathway Analysis, QIAGEN Inc., https://www.qiagenbioinformatics.com/products/ingenuitypathway-analysis)^19^ to examine the crosstalk between the *TGFB*1 signaling pathway and miR-34a and miR-16a-5p (seed of miR-424-5p). We revealed a number of genes and pathways, most notably *VEGFA*, that are reciprocally regulated by these negatively correlated transcription regulators. (**B**) Total mRNA was purified from CCs denuded from GV COC and M2 COC aspirated during IVF procedures. The mRNAs were subjected to qPCR in duplicate with the examined genes and ACTB primers. Gene expression was calculated relative to the ACTB level in the same sample and expression levels were compared using Student’s t-test. The difference reached p = 0.006 for FOXM1, p = 0.01 for TOP2A and p = 0.08 for CD47. RNAseq (green) and qPCR (blue) results are presented as log2-fold change between COC M2 and COC GV samples.

Also noted was the reciprocal crosstalk between *TGFB1* (inhibited), miR-16-5p and miR-34a-5p (both activated) signaling (Fig. 4). All three transcription regulators inhibit *VEGFA* expression observed during the ovulatory process. *TGFB1* (inhibited) activates *E2F1*, while miR-34a (activated) inhibits *E2F1* signaling to the net effect of *E2F1* inhibition. *TGFB1* also inhibits mir-34a.

Interestingly, both upstream regulators, miR-16-5p (seed sequence of miR-424-5p) and miR-34a-5p, are also upregulated in granulosa cells during ovulation (Table 2 and Fig. 1C).

### Functional validation of upstream regulator analysis results

We chose several of the putative miRNA target genes and regulators that were identified by the upstream regulator analysis and performed qPCR analysis. In CCM2 cells, *FOXM1* and *TOP2A* were downregulated, whereas miR-21 was upregulated (Figs 3C and 4B, fold induction). Additionally, miR-34a-5p was upregulated, while *TOP2A* and CD47 were downregulated (Fig. 4, fold induction).

## Discussion

A number of previous studies have examined mRNA and/or miRNA expression in cumulus granulosa cells. However, no study has examined global miRNA expression in human cumulus granulosa cells during final follicular maturation and ovulation. Moreover, this is the first study to link the miRNA-target gene pairs with an inverse correlation of expression. This approach allows the identification of the regulated mRNA-miRNA networks during final follicular maturation and ovulation.

Differential expression analysis revealed dramatic changes in miRNA expression during the CC maturation process; all differentially expressed miRNAs were up-regulated in CCM2 cells.

Compared to published miRNA expression in CCs obtained from M2 COC during IVF treatment^8^, we observed that 8 of the 10 most abundant miRNAs were also present in the CCM2 sample and 43 of the 46 expressed miRNAs were identified by Velthut-Meikas *et al*.

Forty-two of the differentially expressed miRNAs were previously identified in granulosa cells. Among them, 14 are involved in steroidogenesis or PCOS, 14 are involved in apoptosis (eight of which belong to the let-7 family), 12 have been implicated in bovine follicular development and 20 were described as differentially expressed between human cumulus and mural granulosa cells. miR-4454 was not previously described in granulosa cells and was recently implicated in the pathogenesis of cartilage degeneration by promoting inflammatory, catabolic, and cell death activity in chondrocytes^21^. We believe that miR-4454 is implicated in the control of final follicular maturation perhaps by promoting similar proinflammatory-like activities in granulosa cells.

The differentially expressed miRNAs were predicted to regulate 696 target genes that were differentially expressed in our previously generated ovulatory cDNA library, of which, 234 displayed an inverse correlation.

During the final stages of ovulation, several upregulated processes were previously identified by the analysis of global mRNA expression, such as cellular movement, inflammatory response, immune cell trafficking, tissue development and lipid metabolism^3,22,23^. However, several processes, such as tissue morphology, DNA replication and cell cycle, are downregulated^3,22,23^. These downregulated processes were also represented in our analyses of miRNA targets. We observed that a large number of the putative miRNA target genes are related to cell cycle and DNA replication processes. These are probably downstream to the FSH blocking effect following the LH surge, thus leading to decreased proliferation^24^. Interestingly, all of the differentially expressed miRNAs were upregulated in our analysis. This result suggests that the main role of miRNA in the late ovulatory process is more toward control and inhibition of granulosa cell proliferation and less toward other processes found when analyzing gene, rather than miRNA, expression.

The top identified pathways among the miRNA negatively correlated targets include several pathways that pertain to cellular proliferation and survival, including “RB in cancer”, “E2F regulation of DNA replication” and “cell cycle”. Among them, the retinoblastoma protein (RB) was identified as an important factor in ovarian physiology. Conditional deletion of the RB gene in murine ovarian granulosa cells leads to increased follicular recruitment and premature ovarian failure^25^.

We also identified the O-glycosylation of proteins pathway, which might pertain to cumulus matrix maintenance since cumulus complexes from O-glycan-deficient oocytes were smaller and contained fewer CCs, although fertility was not impaired^26^.

Several pathways involved in ovulation were identified in our analyses, including the ERK signaling pathway^27^, HGF signaling^28^, MAPK pathway^27^, P70-S6K signaling^29^, neurotropin TRK signaling^30^, FGF receptor signaling^31^, EGF receptor signaling^31^, TGFB receptor signaling^32^, WNT signaling^33^, VEGF signaling^34^, insulin receptor signaling^35^, and RAS signaling^36^. These findings strengthen and validate our miRNA results.

As expected, more potential novel pathways, including NFAT (nuclear factor of activated T-cells) and PAK (p21-activated kinase) signaling pathways, were found in the broader analysis, which included all miRNA targets (and not only the anti-correlated ones).

NFAT has not been described in granulosa cells, although it has been implicated in GnRH signaling^37^. NFAT was also described as a regulator of *COX2* in endometrial stromal cells^38^ and other tissues and may play a role in prostaglandin regulation during ovulation.

PAK signaling was implicated in cell migration by altering actin cytoskeletal dynamics downstream to Rac/Cdc42, although this activity was not described in granulosa cells and may be important during cumulus expansion and ovulation^39^.

miR-21 is highly expressed and upregulated by LH in murine granulosa cells^40^ and ovine follicles^12^. miR-21 suppression results in granulosa cell apoptosis^40^. Our results further corroborate miR-21 upregulation in humans during the ovulatory process and, by combining our negatively correlated ovulatory gene expression data, we suggest a mechanism of action for miR-21 function in granulosa cells. We found 10 different transcripts regulated by miR-21 that were downregulated in our library, all of which are involved in the cell cycle and apoptosis. These findings expand our knowledge of the processes that lead to the observed inhibition of proliferation on granulosa cells during the final stages of the ovulatory process. Some of the targets were already identified in granulosa cells, including *CDC25a* (apoptosis, cell cycle^41^), *MSH2* (apoptosis^42^), *PRC1* (apoptosis^43^) and *STMN1* (cell migration, apoptosis^44^). Other targets that were not previously described in granulosa cells include *TOP2A* (apoptosis, cell cycle), *DDAH1* (apoptosis), *ECT2* (cell cycle), *RAD51AP1* (cell cycle), *PBK* (apoptosis) and *NUSAP1* (cell migration, apoptosis). The known and previously unknown targets provide insight into miR-21 activity in granulosa cells. The negative correlation between *TOP2A* and miR-21 was experimentally validated (Figs 1C and 4B).

Forkhead box M1 (*FOXM1*), a member of the large family of Forkhead box transcription factors, is highly expressed in proliferating cells and plays pivotal roles in embryonic and fetal development, DNA replication and mitosis^45^. The role of *FOXM1* in ovulation and granulosa cells has not yet been established. *FOXM1* is downregulated in granulosa cells obtained from obese patients undergoing IVF compared to lean patients^46^ and was also found as an inhibited upstream regulator of negatively correlated miRNA target genes in our analysis. Our observation suggests that the reciprocal activation of miR-21 and suppression of *FOXM1* synergize to cause the observed inhibition of granulosa cell proliferation during the final follicular maturation just prior to ovulation. Interestingly, high expression of miR-21 is implicated in increased proliferation in cancer and inflammation^47^. However, the inhibition of breast cancer cell growth by 3, 3′-diindolylmethane is mediated by the upregulation of miR-21 and *CDC25A* and *FOXM1* suppression^48^, consistent with that observed in cumulus cells during ovulation^3^. These *in silico* findings between *FOXM1*, *TOP2A*, and miR-21 were experimentally validated (Figs 1C and 4B).

The let-7 family of miRNAs is believed to act as tumor suppressors^49^ and has been implicated in granulosa cell atresia in porcine^50^. This family was among the most upregulated miRNAs during final follicular maturation. Several of its negatively correlated targets were previously identified in granulosa cells, including *AURKB* (cell cycle^51^), *CDC25A* (apoptosis, cell cycle^41^), *EZH2* (histone methylation^52^), and *THBS1* (adhesion, angiogenesis^53^). Other targets that were not described in granulosa cells include *BRCA2* (DNA repair), *CDC6* (DNA replication), *FANCD2* (DNA repair), *MCM3* (DNA replication), *PPP1R12B* (myosin phosphatase), *RRM2* (DNA synthesis) and *CHEK1* (cell cycle). Most negatively correlated let-7 targets are related to DNA replication/repair and cell cycle progression. Thus, the putative role of let-7 upregulation during final follicular maturation may be in mediating the observed inhibition of granulosa cell proliferation.

We determined that miR-34a is upregulated in CCM2 compared to CCGV. miR-34a is a tumor suppressor gene that suppresses ovarian cancer proliferation and motility by targeting AXL receptor tyrosine kinase^54^. miR-34a levels are increased in sheep granulosa cells during the follicular to luteal transition^12^. In addition, miR-34a may be involved in granulosa cell apoptosis in pig ovaries by targeting the inhibin B gene^55^. The integrated mRNA-miRNA analysis suggests that miR-34a acts through the regulation of *E2F1* (transcription), *CD47* (adhesion), *MCM3* (proliferation), *VEGFA* (angiogenesis) and *TGFB1* signaling during final follicular maturation and luteinization.

Furthermore, the roles of the TGFB1 signaling pathways in folliculogenesis have been extensively studied^56^. The Ingenuity’s IPA upstream regulator analytic identified 38 inhibited *TGFB1* targets of the 234 putative miRNA negatively correlated targets, making it the most significant miRNA-regulated pathway. When *TGFB1* targets were plotted along with miR-16-5p (seed of miRNA-424-3p) and miR-34a targets, we determined that *VEGFA* expression was affected by all of these regulators. It was previously shown by us^3^ and others^2^ that *VEGFA* expression is reduced just prior to ovulation and increased in the luteal phase. This identified regulatory network suggests a novel cooperative mechanism for *VEGF* transient downregulation.

In conclusion, integrated analysis between the expression of coding genes from our preliminary study with the newly generated library of regulatory miRNAs enabled us to better understand the regulation of coding ovulatory genes by non-coding transcripts and their function from the single-molecule level to whole pathways.

## Methods

### Ethical approval

The current study was approved by the Sheba Medical Center Institutional Review Board (IRB) Committee (ethical approval number 8707-11-SMC), and written informed consent was obtained from each patient.

All experiments were performed in accordance with relevant guidelines and regulations.

### IVF protocol

Ovarian stimulation was carried out as previously described^57,58^ according to the “short antagonist” protocol. The protocol consisted of controlled ovarian hyperstimulation with recombinant FSH (rFSH), either Gonal-F; Merck Serono or Puregon Pen; Schering Plough) and human menopausal gonadotropin (HMG; Menopur; Ferring) followed by the addition of ovarian suppression with GnRH antagonists (0.25 mg/day, Cetrorelix, Cetrotide; Serono International, SR) when the leading follicle was more than 12 mm in diameter. When three or more follicles exceeded 18 mm in diameter, 250 µg of hCG (Ovitrelle; Merck Serono) was administered to trigger ovulation. Transvaginal follicular aspiration was performed 35 hours later with ultrasound guidance.

For NanoString analysis, three women (ages 25–35 years) undergoing IVF donated cumulus cells from one M2 COC (see Supp. Table S1 for details).

For the miRNA NanoString validation experiments by qPCR, cumulus cells were obtained from 3–4 different women and pooled to generate a single replicate. Each woman donated CGCs from one M2 COC. The women’s ages and infertility etiologies were similar to those described in Table S1.

For functional validation of upstream regulator analysis, cumulus cells were obtained from 3–4 different women and pooled to generate a single replicate. Each woman (total −21 women) donated CGCs of one M2 or one GV COC. Due to the limited availability of GV COC obtained from IVM, we used cumulus cells of GV COC obtained during IVF. The age of the women was 28–40 years (average 36.5 years). The etiology of infertility included male factor and PGD.

### IVM protocol

Four women (ages 28–40 years) undergoing IVM were selected for this study (see Supp. Table S1 for details). In the final analysis, only 3 women were included; one woman (Patient code 59 A in Table S1) was excluded because she was an outlier. Each woman donated one single COC. IVM cycles were carried out as previously described^59^. Briefly, sonographic assessment of the antral follicle count and of endometrial thickness was carried out on day 3 of a spontaneous menstrual cycle. The serum concentrations of estradiol and progesterone were also determined. Next, 150 IU/day rFSH were administered to the patients for 3 days. A second evaluation was performed on day 6. An injection of 10000 IU hCG (Pregnyl; Organon, Oss, Holland) was administered subcutaneously when the endometrial thickness was ≥5 mm and the leading follicle was at least 12 mm. Oocyte retrieval was carried out 36 hours later. Compact CCs were obtained from surplus germinal vesicle (GV) oocytes that were acquired during IVM treatment (CCGV group, provided by Dr. Ruben Fadini from the Biogenesi Reproductive Medicine Center, Istituti Clinici Zucchi, Monza, Italy).

For NanoString analysis, four women (ages 28–40 years) undergoing IVM were selected (see Supp. Table S1 for details). In the final analysis, only 3 women were included; one woman (Patient code 59 A in Table S1) was excluded because she was an outlier. Each woman donated one single COC. For the miRNA NanoString validation experiments, cumulus cells were obtained from 3–4 different women and pooled to generate a single replicate. Each woman donated CGCs from one GV COC. The women’s ages and infertility etiologies were similar to those described in Table S1.

### Cumulus granulosa cell collection

CGCs were obtained through oocyte denudation in the course of intracytoplasmic sperm injection (ICSI) procedures. After oocyte retrieval, CGCs of each oocyte were removed using hyaluronidase (SAGE) and a glass denudation pipette (Swemed). The CGCs were washed in PBS and centrifuged at 5000 × g for 5 minutes at room temperature. The resulting pellets were stored at −80 °C until RNA isolation.

### RNA extraction

Total RNA was extracted from seven independent CCs samples of seven different women obtained from compact GV (4 samples) and expanded M2 of single COC (3 samples) using a Micro RNA Isolation Kit (Zymo Research Corp., CA USA) according to the manufacturer’s instructions. RNA purity and concentration were assessed using a NanoDrop spectrophotometer (NanoDrop 2000C, Thermo Fisher Scientific).

### Quantitative real-time PCR

cDNA synthesis for the detection of mature miRNAs was performed with the miScript Reverse Transcription Kit (QIAGEN) using a blend of oligo(dT) and random primers (iScript™ cDNA Synthesis Kit). cDNA synthesis for the quantification of mRNA expression was performed with the High Capacity cDNA Reverse Transcription Kit (Applied Biosystems). miRNA expression was determined using the miScript SYBR Green Kit (QIAGEN) and mRNA expression was determined using Fast SYBR Green Master Mix (Applied Biosystems). The StepOnePlus Real-Time PCR System (Applied Biosystems) was used to detect amplification.

Expression levels were normalized to ACTB (for mRNA) and RNU6B (for miRNA). qPCR results were analyzed with StepOne software. Relative gene expression was calculated using the delta-delta Ct method^60^. Details of the primers are shown in Table S2.

### NanoString and bioinformatics sample analysis

Evaluation of miRNA expression was carried out using NanoString technology, which is not based on sequencing but rather based on a digital molecular barcoding system. Each barcode is attached to a single target-specific probe corresponding to a specific miRNA. The output from this technology is the digital count of 800 tested miRNAs.

A total of 3 μl (20–120 ng) of each RNA sample was prepared according to the manufacturer’s instructions. Mature miRNAs were ligated to a species-specific tag sequence (miRtag) via a thermally controlled splinted ligation. miRNA assays were performed according to the NanoString miRNA Assay Manual. Hybridizations were carried out by combining 5 µl of each miRNA assay with 20 µl of nCounter Reporter probes in hybridization buffer and 5 µl of nCounter Capture probes for a total reaction volume of 30 µl. miRNA expression was assessed using the NanoString nCounter system (NanoString Technologies, Seattle, WA, USA), which enables multiplexed direct digital counting of 800 human miRNA molecules. The raw count data from the 6 samples were analyzed using the edgeR^61^ and limma R^62^ packages. First, TMM normalization was applied to the data followed by voom transformation. To assess the number of miRNAs expressed under each condition, we required that probes be detectable in all replicates of the tested condition. As detectable, we defined probes having a normalized expression level that exceeded that of the “highest expressed” negative control probe. This resulted in 54 expressed miRNAs. Next, linear models were used to assess differential expression using the limma method. Pairwise comparisons were performed using moderated t statistics. To perform multiple group comparisons, one-way ANOVA was applied, except the residual mean squares were moderated between genes (see the limma package for details^62^). miRNAs were considered differentially expressed if their FDR was <5%.

The datasets generated during and/or analyzed during the current study are available from the corresponding author upon reasonable request.

### Bioinformatics analysis of the library

The resultant differentially expressed miRNAs were analyzed through the use of IPA (Ingenuity Pathway Analysis QIAGEN Inc. https://www.qiagenbioinformatics.com/products/ingenuitypathway-analysis)^19^. Putative miRNA targets found using Ingenuity miRNA target filter for experimentally validated and putative predicted targets (high confidence level) – *in silico* analysis.

Using whole transcriptome sequencing, we previously generated an mRNA ovulatory genes library^3^. The library was comprised of the same groups as in the current study (CCGV and CCM2), encompassing all differentially expressed mRNAs between immature mid-antral follicles and mature pre-ovulatory follicles.

Our previously generated mRNA library and the miRNA putative targets were combined using the microRNA target filter to generate a subset of the negatively correlated miRNA targets – experimental analysis.

Both mRNA target lists (*in silico* analysis target list and experimental analysis target list) were analyzed for GO terms and pathways using the GeneAnalytics tool^20^.

The DE miRNAs and negatively correlated targets were also analyzed using the Ingenuity Upstream Regulator Analysis tool to predict upstream molecules, including miRNA and transcription factors, that may have caused the observed gene expression changes.

### Statistics

Each experiment was performed at least three times. Data are expressed as the mean ± standard error of the mean (SEM) and were evaluated using Student’s t-test with a two-tailed distribution, with two samples equaling variance, or with ANOVA (ANalysis Of VAriance) for more than two variances using the post hoc Tukey test assuming equal variance, or the Games–Howell test for unequal variance. When appropriate, the Kruskal–Wallis non-parametric comparison test was used. For all statistical analyses, SPSS 22 software (IBM, Armonk, NY, USA) was used. P-value < 0.05 was considered statistically significant.

## Electronic supplementary material

Table S1 and Table S2

Table S3

Table S4

Table S5

Table S6

Table S7

Table S8

Table S9
