# Supplementary material for: Characterization of the miRNA regulators of the human ovulatory cascade
Source: Sci Rep. 2018 Oct 23;8:15605. doi: 10.1038/s41598-018-33807-y (PMC6199329; doi:10.1038/s41598-018-33807-y)
Supplement: Supplementary file 1 — Table S1 and Table S2 [file 41598_2018_33807_MOESM1_ESM.docx]

**Characterization of the miRNA regulators of the human ovulatory cascade**

G.M. Yerushalmi, M. Salmon-Divon, L. Ophir, Y. Yung, M. Baum, G. Coticchio, R. Fadini, M. Mignini Renzini, M. Dal Canto, R. Machtinger, E. Maman, A. Hourvitz

**Supplementary Materials**

**Table S1.** Clinical characteristics of patients enrolled for this study.

**Table S2**. miRNA and mRNA primers used in this study**.**

**Table S3**. Differently Expressed miRNA Target Genes (predicted + experimentally validated) identified by QIAGEN’s Ingenuity Pathway Analysis software[^19^](#_ENREF_19).

**Table S4**. Differently Expressed miRNA Target Genes (experimentally validated) identified by QIAGEN’s Ingenuity Pathway Analysis software[^19^](#_ENREF_19).

**Table S5.** GeneAnalytics[^20^](#_ENREF_20) - GO-Biological Process of unfiltered miRNA targets (7244 genes).

**Table S6**. GeneAnalytics[^20^](#_ENREF_20) - Pathway results of unfiltered miRNA targets (7244 genes).

**Table S7.** GeneAnalytics[^20^](#_ENREF_20) - GO-Biological Process of anti-correlated miRNA targets (234 genes).

**Table S8**. GeneAnalytics[^20^](#_ENREF_20) - Pathway results of anti-correlated miRNA targets (234 genes).

**Table S9**. Significant upstream regulators for anti-correlated miRNA and targets identified by ingenuity IPA analysis (Z-score greater than ±2)[^19^](#_ENREF_19).

**Table S1** Clinical characteristics of patients enrolled for this study.

| Patient code | Treatment | Age | Infertility | No of oocytes retrieved |
| --- | --- | --- | --- | --- |
| CCGV-3A | IVM | 36 | Male Factor | 14 |
| CCGV-24A | IVM | 40 | Male Factor | 15 |
| CCGV-61A | IVM | 28 | PCOS | 14 |
| CCGV-59A* | IVM | 37 | Male Factor | 17 |
| CCM2-3127 | IVF | 30 | Male Factor | 8 |
| CCM2-3210 | IVF | 25 | PGD | 11 |
| CCM2-3127 | IVF | 30 | Male Factor | 15 |

*excluded from the final nanostring analysis

**Table S2** miRNA and mRNA primers used in this study

| miRNA | Primer sequence |
| --- | --- |
| hsa-miR-424-5p | CGCAGCAGCAATTCATGTTTTGAA |
| hsa-miR-34a-5p | CTGGCAGTGTCTTAGCTGGTTGT |
| hsa-miR-21 | GCGTAGCTTATCAGACTGATGTTGA |
|  |  |
| mRNA |  |
| FOXM1_F | AGAAGTGACCCTGGAGACCT |
| FOXM1_R | CCTGCTGCCTCACCATCTG |
| TOP2A_F | GGCTACATGGTGGCAAGGAT |
| TOP2A_R | CATTCAGGCTCAACACGCTG |
| CD47_F | TACCAATGCATGGCCCTCTT |
| CD47_R | CTTCAGTTATTCCTAGGAGGTTGT |
